# A human liver chimeric mouse model for non-alcoholic fatty liver disease

**DOI:** 10.1016/j.jhepr.2021.100281

**Published:** 2021-03-21

**Authors:** Beatrice Bissig-Choisat, Michele Alves-Bezerra, Barry Zorman, Scott A. Ochsner, Mercedes Barzi, Xavier Legras, Diane Yang, Malgorzata Borowiak, Adam M. Dean, Robert B. York, N. Thao N. Galvan, John Goss, William R. Lagor, David D. Moore, David E. Cohen, Neil J. McKenna, Pavel Sumazin, Karl-Dimiter Bissig

**Affiliations:** 1 Department of Pediatrics, Division of Medical Genetics, Duke University, Durham, NC, USA; 2 Department of Molecular Physiology and Biophysics, Baylor College of Medicine, Houston, TX, USA; 3 Texas Children’s Cancer Center, Department of Pediatrics, Baylor College of Medicine, Houston, TX, USA; 4 Department of Molecular and Cellular Biology, Baylor College of Medicine, Houston, TX, USA; 5 Institute for Molecular Biology and Biotechnology, Faculty of Biology, Adam Mickiewicz Universtiy, Poznan, Poland; 6 Department of Surgery, Texas Children’s Hospital, Houston, TX, USA; 7 Joan & Sanford I. Weill Department of Medicine, Weill Cornell Medical College, New York, NY, USA; 8 Y.T. and Alice Chen Pediatric Genetics and Genomics Research Center, Duke University, Durham, NC, USA; 9 Division of Gastroenterology, Department of Medicine, Duke University, Durham, NC, USA; 10 Department of Pharmacology and Cancer Biology, Duke University, Durham, NC, USA; 11 Duke Cancer Institute, Duke University, Durham, NC, USA

**Keywords:** Non-alcoholic fatty liver disease, Steatosis, Lipid metabolism, Human disease modelling, Humanised mice, ALP, alkaline phosphatase, ALT, alanine aminotransferase, AST, aspartate aminotransferase, CBPEGs, cholesterol biosynthesis pathway enzyme genes, CE, cholesteryl ester, CER, ceramide, CHHs, chimeric human hepatocytes, CMHs, chimeric mouse hepatocytes, CT, confidence transcript, DAG, diacylglycerol, DCER, dihydroceramide, DEG, differentially expressed gene, FA, fatty acid, FAH, fumarylacetoacetate hydrolase, FFA, free fatty acid, GGT, gamma-glutamyl transpeptidase, hALB, human albumin, HCC, hepatocellular carcinoma, HCER, hexosylceramide, HCT, high confidence transcriptional target, LCER, lactosylceramide, LPC, lysophosphatidylcholine, LPE, lysophosphatidylethanolamine, MAG, monoacylglycerol, MUFA, monounsaturated fatty acid, NAFLD, non-alcoholic fatty liver disease, NASH, non-alcoholic steatohepatitis, NC, normal chow, NTBC, nitisinone, PC, phosphatidylcholine, PE, phosphatidylethanolamine, PI, phosphatidylinositol, PNPLA3, patatin-like-phospholipase domain-containing protein 3, PUFA, polyunsaturated free FA, SM, sphingomyelin, SREBP, sterol regulatory element-binding protein, TAG, triacylglycerol, TIRF, transgene-free *Il2rg*^-/-^/*Rag2*^-/-^/*Fah*^-/-^, WD, Western-type diet

## Abstract

**Background & Aims:**

The accumulation of neutral lipids within hepatocytes underlies non-alcoholic fatty liver disease (NAFLD), which affects a quarter of the world’s population and is associated with hepatitis, cirrhosis, and hepatocellular carcinoma. Despite insights gained from both human and animal studies, our understanding of NAFLD pathogenesis remains limited. To better study the molecular changes driving the condition we aimed to generate a humanised NAFLD mouse model.

**Methods:**

We generated TIRF (transgene-free *Il2rg*^-/-^/*Rag2*^-/-^/*Fah*^-/-^) mice, populated their livers with human hepatocytes, and fed them a Western-type diet for 12 weeks.

**Results:**

Within the same chimeric liver, human hepatocytes developed pronounced steatosis whereas murine hepatocytes remained normal. Unbiased metabolomics and lipidomics revealed signatures of clinical NAFLD. Transcriptomic analyses showed that molecular responses diverged sharply between murine and human hepatocytes, demonstrating stark species differences in liver function. Regulatory network analysis indicated close agreement between our model and clinical NAFLD with respect to transcriptional control of cholesterol biosynthesis.

**Conclusions:**

These NAFLD xenograft mice reveal an unexpected degree of evolutionary divergence in food metabolism and offer a physiologically relevant, experimentally tractable model for studying the pathogenic changes invoked by steatosis.

**Lay summary:**

Fatty liver disease is an emerging health problem, and as there are no good experimental animal models, our understanding of the condition is poor. We here describe a novel humanised mouse system and compare it with clinical data. The results reveal that the human cells in the mouse liver develop fatty liver disease upon a Western-style fatty diet, whereas the mouse cells appear normal. The molecular signature (expression profiles) of the human cells are distinct from the mouse cells and metabolic analysis of the humanised livers mimic the ones observed in humans with fatty liver. This novel humanised mouse system can be used to study human fatty liver disease.

## Highlights

• Human liver chimeric mice fed a western diet develop NAFLD.
• Within the same chimeric liver, human hepatocytes developed pronounced steatosis while murine hepatocytes remained normal.
• Unbiased metabolomics and lipidomics of fatty humanised mouse livers revealed signatures of clinical NAFLD.
• Transcriptomic analyses showed that molecular responses diverged sharply between murine and human hepatocytes.
• This humanised NAFLD model is a physiologically relevant, experimentally tractable model for the study of steatosis.

## Graphical abstract

**Figure:**
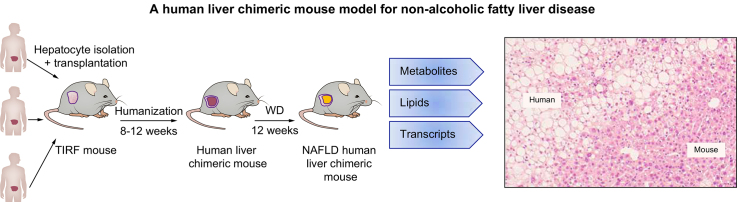

## Introduction

The global spread of high-fat, high-sugar diets and sedentary lifestyles carries with it a sharp rise in non-alcoholic fatty liver disease (NAFLD).^1^ Hepatic steatosis is considered problematic because it leads to inflammation and fibrosis, creating the more serious condition known as non-alcoholic steatohepatitis (NASH).^2^^,^^3^ NASH in turn triggers compensatory cell proliferation, increasing the risk of hepatocellular carcinoma (HCC), which has become the second leading cause of cancer deaths.^4^ Given that steatosis seems to drive pathogenesis in all these major diseases, there is a pressing need to understand how it changes liver function.

NAFLD has been extremely difficult to study in either human patients or mice. Although transcriptomic, lipidomic, and metabolic profiling studies of human livers are numerous, clinical approaches are generally retrospective and human participants are not experimentally tractable. Studies using murine models present different limitations: genetic models of NAFLD based on single-gene alterations, such as the *ob*/*ob* or *db*/*db* obese mouse strains, may not accurately reflect the typically polygenic human situation.^5^ Dietary models gain weight, develop steatosis, and become insulin-resistant, but exhibit much less liver injury than human NASH patients, suggesting fundamental differences in rodent and human livers.[5], [48] Indeed, metabolic profiling of rats fed a methionine-choline-deficient diet—which inhibits fatty acid oxidation, thereby leading to NASH—identified species differences in bile acid, insulin, and leptin levels,^6^ limiting this model’s utility for studies of biomarkers or disease progression.

Having recently developed human liver chimeric mice to study human drug metabolism,^7^ we took a similar approach here to study NAFLD. We demonstrate that humanised TIRF (trangene-free, *Il2rg*^-/-^, *Rag2*^-/-^, *Fah*^-/-^) mice develop NAFLD when fed a Western-type high fat/high sucrose diet (WD). Metabolic and histological changes of human hepatocytes in xenograft mice resemble those found in human NAFLD, whereas the molecular signatures of the human and murine hepatocytes within the same chimeric livers differ greatly. This chimeric mouse model therefore offers an ideal platform for studying the response of human hepatocytes to diet-induced NAFLD.

## Materials and methods

### Animals and diets

The TIRF strain was generated by CRISPR/Cas9 deletion of the 3 genes in mouse zygotes as we described previously^7^ and crossing out the P450 oxidoreductase (*Por*) gene from the PIRF (*Por*^-/-^ *Il2rg*^-/-^, *Rag2*^-/-^, *Fah*^-/-^) strain.^7^

Human hepatocytes (1 × 10^6^ per mouse) were transplanted by splenic injection into the livers of 3-week-old male and female TIRF mice maintained on the drug nitisinone (NTBC) from the drinking water as previously described.^7^^,^^8^ Immediately after transplantation, we applied selection pressure toward transplanted chimeric human hepatocytes (CHHs) by step-wise reduction and withdrawal of NTBC.^7^^,^^8^ human albumin (hALB) levels (ELISA, Bethyl Laboratories, Inc., Montgomery, TX, USA) in the plasma of transplanted mice was measured to gauge the extent of chimerism.^9^ Only mice with >70% human liver chimerism were used and put on NTBC (no tyrosinemic stress of murine hepatocytes) for the remainder of the study.

Humanised mice were fed normal chow (NC) (2019 Teklad global 19% protein extruded rodent diet, Envigo, Madison, Wisconsin USA) or WD (D12079B, RD Western Diet, Research Diets, New Brunswick, New Jersey, USA) for 12 weeks. Mice were euthanised after a 6-h fast and plasma was collected. Livers were then harvested and snap-frozen in liquid nitrogen and stored at -80°C. All animal experiments were approved by the Institutional Animal Care and Use Committee. Animals received humane care according to the criteria outlined in the NIH Guide for the Care and Use of Laboratory Animals.

### Metabolomic and lipidomic analyses

Sample preparation and analysis for global metabolomics was carried out at Metabolon Inc. (Durham, NC, USA) as described by Hatori *et al.*,^10^ with modifications (see Supplementary Methods).

Lipids for untargeted lipidomics were extracted from snap-frozen liver samples at Metabolon Inc. using dichloromethane and methanol in the presence of deuterated internal standards. Details for extraction and analysis can be found in the Supplementary Methods.

### RNA-sequencing, data processing, and NAFLD consensome analysis

Whole-transcriptome RNA-sequencing (RNA-Seq) was performed using total RNA isolated using a Purelink RNA mini kit (Invitrogen PureLink RNA Mini Kit, ThermoFisher Scientific, USA). RNA-seq read pairs were aligned to a combined human (hg38) and murine (mm10) genome using STAR version 2.7.1a. RNA-Seq data is available from European Nucleotide Archive, ENA accession code (PRJEB35014). See Supplementary Methods for details of library generation and gene expression quantification.

To generate the human NAFLD consensome, we first retrieved a total of 9 Gene Expression Omnibus clinical NAFLD or NASH case-control transcriptomics datasets (Table S6, section 3) (see Supplementary Methods). Differential expression values were committed to the consensome analysis pipeline as previously described.^11^ Panther^12^ and Reactome pathway^13^ analysis was used to evaluate enrichment of biological processes in NAFLD consensome confidence transcripts (CTs) or H:M>1.2 genes. Node high confidence transcriptional target (HCT) intersection analysis was performed as previously described.^14^

Additional methods are described in the Supplementary Methods.

## Results

### Human liver chimeric mice develop steatosis after 12 weeks on a WD

To evaluate the susceptibility of xenograft mice to diet-induced NAFLD, we developed the TIRF strain. In contrast to previous *Fah*^*-/-*^*/Rag2*^*-/-*^*/Il2rg*^*-/-*^ mice,^15^^,^^16^ this strain has no neomycin-resistance cassettes that could confound results in several ways. The neomycin phosphotransferase gene can influence the metabolism of transgenic mice^17^; the transgene can alter gene expression and has been shown to increase expression of both c-myc and various liver genes^18^; and it was shown to reduce fructose 2,6-bisphosphate and lactate in fibroblasts.^18^

**Fig 1:**
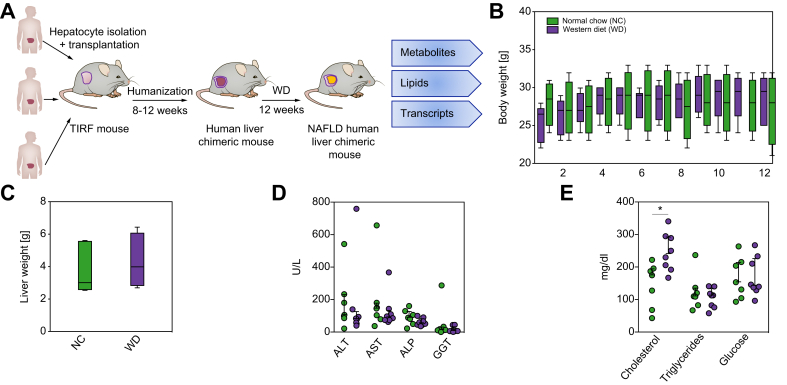
Experimental set-up and basic parameters of humanised TIRF mice on Western-type diet (WD). (A) TIRF mice were transplanted with 3 different samples of human hepatocytes. After reaching high human chimerism, animals were placed on WD or NC for 12 weeks, after which they were euthanised for transcriptomic, metabolomic, and lipidomic analyses of the human liver cells. (B) Body weights (n = 6–8 per group), (C) liver weights (n = 6–8 per group), and (D,E) blood chemistry (n = 6–8 per group) after 12 weeks. (E) Plasma concentrations of cholesterol, triacylglycerol, and glucose after 12 weeks of diet. Data are presented as mean ± SD. ^∗^*p* <0.05 using Student's *t*-test. ALP, alkaline phosphatase; ALT, alanine aminotransferase; AST, aspartate aminotransferase; GGT, gamma-glutamyl transpeptidase; NAFLD, non-alcoholic fatty liver disease; NC, normal chow; TIRF, transgene-free *Il2rg*^-/-^/*Rag2*^-/-^/*Fah*^-/-^.

Three-week-old male and female TIRF mice were humanised by splenic injection of human hepatocytes from 1 of 3 different healthy human donors (Fig. 1A). Three to 4 months after transplantation, human chimerism was assessed by measurement of hALB in the murine plasma and only mice with >70% human hepatocytes were used in subsequent studies. Mice bearing human hepatocytes were randomised and fed either WD or NC for 12 weeks (Fig. 1A). To mimic low mitotic index of the human liver, we removed selection pressure for human hepatocytes in humanised mice (see Methods for details).

Relative to NC, WD induced no significant change in body or liver weight (Fig. 1B and C), blood chemistries (alanine aminotransferase, aspartate aminotransferase, alkaline phosphatase, and gamma-glutamyl transpeptidase; Fig. 1D) or fasting levels of plasma triacylglycerols or glucose over the 12-week period. Plasma cholesterol, however, increased 70% (Fig. 1E). This is consistent with observations from patients with NAFLD, where intrahepatic fat accumulation occurs before the development of obesity or abnormalities in standard liver function tests.^19^ As no selection pressure for human hepatocytes was applied, we compared hALB levels before and after 12 weeks on WD, respectively, with NC. There was no significant decrease on NC, but WD decreased the human albumin levels from an average of 4.8 mg/ml to 2.2 mg/ml (Fig. S1).

**Fig 2:**
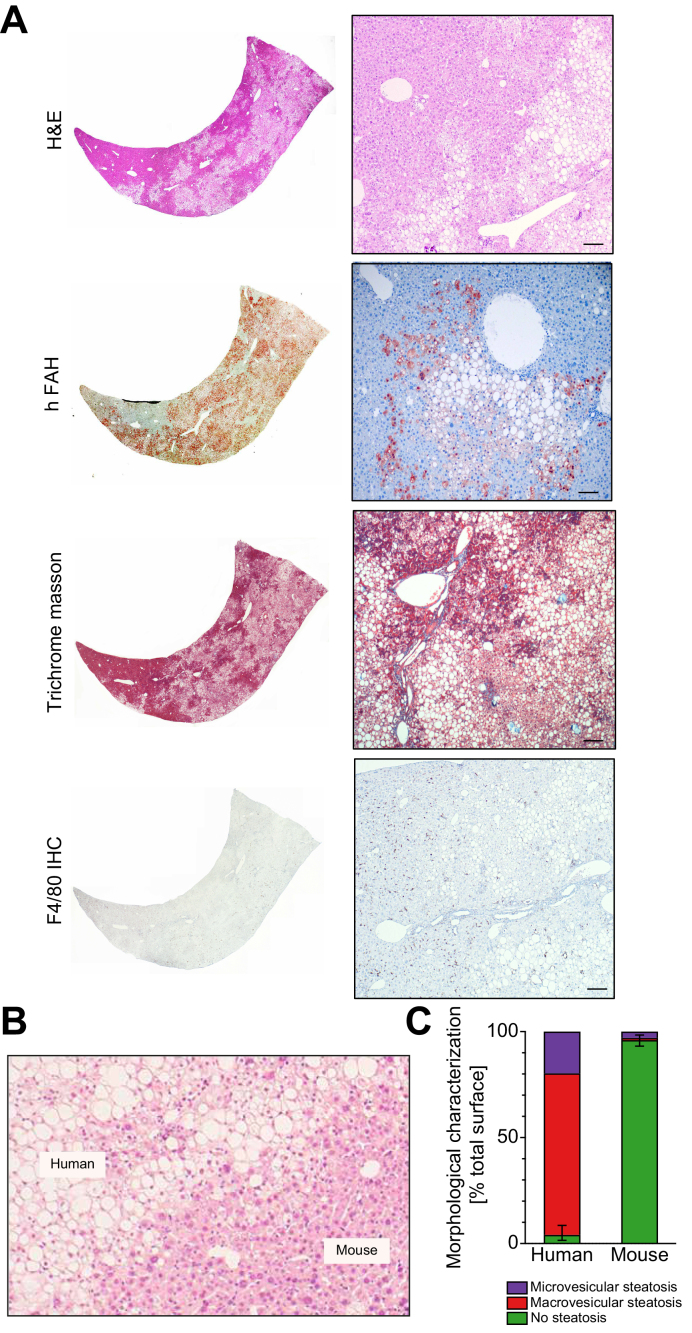
Diet-induced steatosis is associated with human hepatocytes in NAFLD xenograft mice. Histological analyses were performed in liver sections after 12 weeks of Western-type diet (n = 6–8 per group). (A) Representative images of H&E-, fumarylacetoacetate hydrolase (FAH)-, Trichrome Masson-stained and F4/80-immunostained liver sections. Scale bar = 50 μm. (B) Representative image of H&E-stained liver sections showing separation of hepatocytes from human or murine origin (dotted line). Scale bar = 50 μm. (C) Quantification of macro- and microvesicular steatosis in human and murine liver tissue. Percentage surface area of multiple lobes (2–5) of NAFLD xenograft mice (n = 6) are given with whiskers for the range of non-steatotic human or murine hepatocytes (see methods for details). NAFLD, non-alcoholic fatty liver disease.

We next examined histological changes in livers of humanised mice in response to 12 weeks of WD. H&E staining identified pronounced macrovesicular steatosis with microvesicular components (Fig. 2A and B). Immunostaining for fumarylacetoacetate hydrolase (FAH) confirmed that steatosis was confined to human hepatocytes, repopulating across the whole liver acinus (Fig. 2A and C). Oil-Red-O staining further confirmed lipid accumulation (Fig. S2). Staining for connective tissue (Trichrome Masson) and the liver-resident macrophages (Kupffer cells, F4/80), did not show appreciable differences between murine and human regions of the chimeric livers (Fig. 2A).

Collectively, these findings indicate that humanised TIRF mice develop human hepatocyte-specific steatosis after 12 weeks of WD, replicating early clinical NAFLD.

### Distinct hepatic metabolite profiles between NAFLD and control xenograft mice

**Fig 3:**
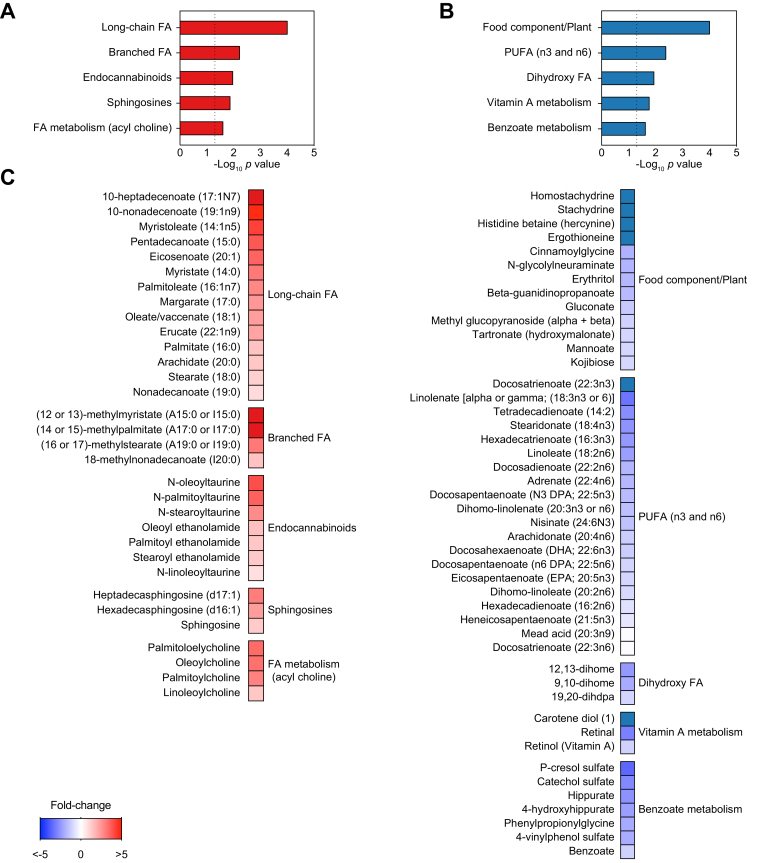
NAFLD xenograft mouse livers show altered metabolic profile after 12 weeks on WD. Global metabolic profiles were determined in livers of 12 week WD or NC chimeric mice (n = 8 per group). (A) Metabolite species increased in livers of NAFLD xenograft mice after 12 weeks of WD. (B) Metabolite species decreased in livers of NAFLD xenograft mice after 12 weeks on WD. (C) Relative levels of individual metabolites are shown. FA, fatty acid; NAFLD, non-alcoholic fatty liver disease; NC, normal chow; PUFA, polyunsaturated fatty acid; WD, Western-type diet.

Because livers of patients with NAFLD undergo profound metabolic changes in comparison with livers of patients without steatotis,^3^ we searched for similar effects in WD-fed humanised mice. Using global metabolomic profiling, we found that among 565 identified metabolites, 48 were significantly increased and 59 were significantly decreased in livers of NAFLD chimeric mice compared with controls (Table 1 and S1). Among metabolites with the largest increases were cholesterol, 4-cholesten-3-one (an intermediate in the synthesis of bile acids) and the antioxidant α-tocopherol (Table 1 and S1). Metabolic pathway enrichment analysis showed WD-induced increases in hepatic concentrations of long-chain and branched free fatty acids (FFAs), intermediates of endocannabinoid, sphingosine, and acyl-choline metabolism (Fig. 3A and C). In contrast, we observed WD-induced decreases in levels of metabolites associated with xenobiotic metabolism, vitamin A, and benzoate (Table 1 and Fig. 3B and C), which have been previously found to be reduced in patients with NAFLD.^3^^,^^20^ Similarly, WD decreased levels of polyunsaturated free fatty acid (PUFA) species (Fig. 3B and C) and dihydroxy fatty acid (FA) lipid peroxide markers (Table 1 and S1). Conversely, the WD induced increases in levels of docosatrienoate and mead acid (Table 1).

**Table 1:** **Top differentially regulated metabolites in NAFLD xenograft mice**. ∗Humanised mice on WD (NAFLD) compared with humanised mice on normal chow (control). NAFLD, non-alcoholic fatty liver disease; WD, Western-type diet.

Metabolite	Pathway	*p* value	Fold change (NAFLD *vs.* control∗)
**Increased**			
Docosatrienoate (22:3n6)	Polyunsaturated fatty acid (n3 and n6)	1.12E-02	12.50
Mead acid (20:3n9)	Polyunsaturated fatty acid (n3 and n6)	4.56E-04	11.95
(14 or 15)-methylpalmitate (a17:0 or i17:0)	Fatty acid, Branched	3.89E-03	10.10
1-oleoyl-GPG (18:1)	Lysophospholipid	8.93E-03	9.18
10-heptadecenoate (17:1n7)	Long chain fatty acid	1.46E-03	5.44
10-nonadecenoate (19:1n9)	Long chain fatty acid	3.41E-06	4.74
Myristoleate (14:1n5)	Long chain fatty acid	1.58E-02	4.24
N-oleoyltaurine	Endocannabinoid	2.99E-02	3.97
Pentadecanoate (15:0)	Long chain fatty acid	1.71E-02	3.76
Eicosenoate (20:1)	Long chain fatty acid	1.64E-04	3.53
Oleoylcholine	Fatty acid metabolism (acyl choline)	2.68E-02	3.26
(16 or 17)-methylstearate (a19:0 or i19:0)	Fatty acid, branched	4.40E-06	3.09
Heptadecasphingosine (d17:1)	Sphingosines	1.29E-03	3.02
Palmitoleate (16:1n7)	Long chain fatty acid	9.18E-04	2.81
1-oleoyl-GPS (18:1)	Lysophospholipid	1.94E-02	2.54
**Decreased**			
Homostachydrine	Food component/Plant	3.64E-04	0.01
Stachydrine	Food component/Plant	3.99E-04	0.02
Docosatrienoate (22:3n3)	Polyunsaturated fatty acid (n3 and n6)	1.46E-05	0.02
Trigonelline (Nʹ-methylnicotinate)	Nicotinate and nicotinamide metabolism	1.30E-03	0.03
Histidine betaine (hercynine)	Food component/Plant	4.89E-05	0.08
4-guanidinobutanoate	Guanidino and acetamido metabolism	9.09E-03	0.10
N,N,N-trimethyl-5-aminovalerate	Lysine metabolism	8.95E-03	0.15
Cis-4-decenoate (10:1n6)	Medium chain fatty acid	9.88E-06	0.17
Carotene diol (1)	Vitamin A metabolism	3.22E-03	0.21
Ergothioneine	Food component/plant	7.38E-05	0.21
13-HODE + 9-HODE	Fatty acid, monohydroxy	1.65E-02	0.21
3-sulfo-L-alanine	Methionine, cysteine, S-adenosylmethionine and taurine metabolism	1.44E-02	0.22
P-cresol glucuronide	Tyrosine metabolism	2.59E-03	0.24
4-hydroxy-nonenal-glutathione	Glutathione metabolism	6.13E-03	0.25
Gamma-tocopherol/beta-tocopherol	Tocopherol metabolism	5.01E-04	0.27

### Humanised mouse livers have lipid profiles similar to those of patients with NAFLD

**Fig 4:**
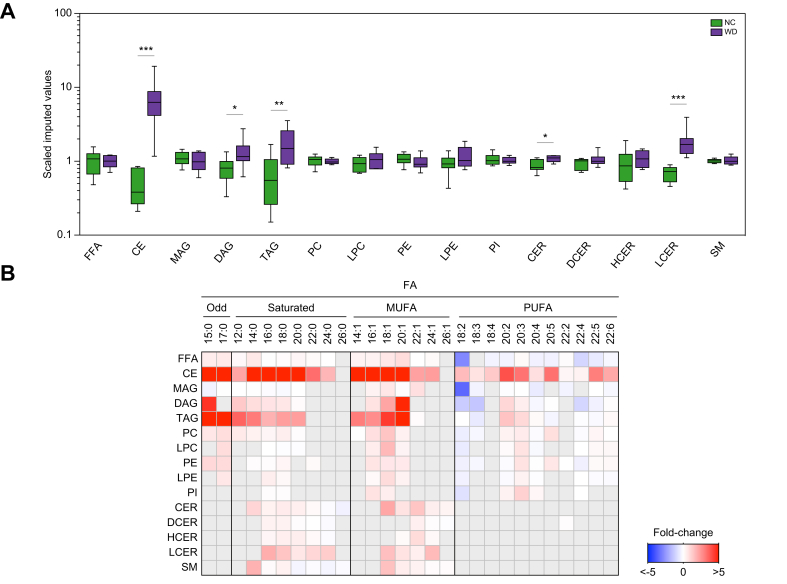
Altered lipid profile in livers of NAFLD xenograft mice. Global lipid profiles were determined in livers of 12-week WD or NC chimeric mice (n = 8 per group). (A) Hepatic levels of lipids. ∗*p* <0.05, ∗*p* <0.01, and ^∗∗∗^*p* <0.001 using Welch's *t*-test. (B) Hepatic fatty acyl composition (relative to NC-fed humanised mice). Grey boxes, data not available. CE, cholesteryl ester; CER, ceramide; DAG, diacylglycerol; DCER, dihydroceramide; FFA, free fatty acid; HCER, hexosylceramide; LCER, lactosylceramide; LPC, lysophosphatidylcholine; LPE, lysophosphatidylethanolamine; MAG, monoacylglycerol; NAFLD, non-alcoholic fatty liver disease; NC, normal chow; PC, phosphatidylcholine; PE, phosphatidylethanolamine; PI, phosphatidylinositol; TAG, triacylglycerol; WD, Western-type diet.

Accumulating evidence suggests that NAFLD progression is associated with changes in hepatic lipid species composition and concentration. The observation that levels of FFA species were altered in livers of WD-fed humanised mice prompted us to evaluate whether this would be reflected in changes in the overall hepatic lipid profile. Although most studies have used targeted analysis,[21], [22], [23], [24], [25], [26] we used untargeted lipidomics and found increased steady-state concentrations of cholesteryl esters (CEs), diacylglycerols (DAGs), triacylglycerols (TAGs), ceramides (CERs), and lactosylceramides (LCERs) in livers of WD-fed chimeric mice in comparison with NC-fed counterparts (Fig. 4A and Table S2).

We next examined the influence of the WD in the composition of FAs that are esterified to complex lipids in livers of xenograft mice. The composition of FFAs as well as FAs in DAG, TAG, and lysophosphatidylethanolamine exhibited increased odd, saturated, and monounsaturated fatty acid (MUFA) species, along with decreased PUFAs (Fig. 4B), resembling livers of patients with NAFLD.^22^^,^^24^^,^^27^ In the chimeric mice, hepatic steatosis coincided with increases in all analysed FA species esterified to CE (Fig. 4B). However, monoacylglycerols (MAGs) and glycerophospholipids showed increased esterification of odd FAs and MUFAs, with an overall decrease in saturated and PUFA species (Fig. 4B), an effect that was more pronounced in n-3 PUFA species (Table S2). Sphingolipids reflected a preference for the esterification of medium- to long-chain saturated FAs and MUFAs, along with decreased esterification of very long-chain saturated FAs (Fig. 4B).

### Mouse and human hepatocytes of NAFLD xenograft mice exhibit different transcriptional profiles

**Fig 5:**
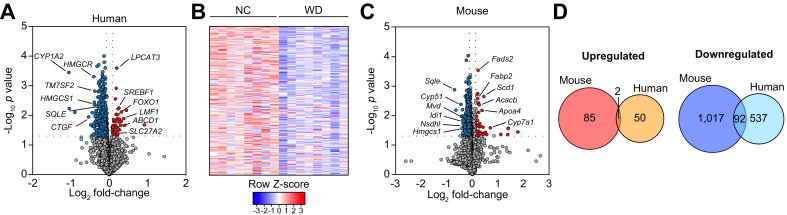
Divergent transcriptional responses to WD between CHHs and CMHs in NAFLD xenograft mice. Transcriptomic analysis of human (A and B) and mouse (C and D) hepatocytes was performed in livers of 12 week WD or NC chimeric mice (n = 8 per group). Upregulated genes in the WD-fed mice relative to the NC group are shown in red, downregulated genes are in blue. (D) Groupwise comparison of differentially expressed mouse and human genes (WD *vs.* NC). CHHs, chimeric human hepatocytes; CMHs, chimeric mouse hepatocytes; NC, normal chow; NAFLD, non-alcoholic fatty liver disease; WD, Western-type diet.

To determine whether changes in hepatic metabolites and lipids could be attributable to altered transcriptional profiles in the NAFLD model, we performed a transcriptional analysis of CHHs in NC- and WD-fed chimeric mice. We identified a total of 681 differentially expressed genes (DEGs) when comparing WD-fed with NC-fed chimeric mice (Fig. 5A and B, Table S3 and S4). Pathway enrichment analysis revealed that upregulated genes (52 genes; 7.6% of total DEGs) were predominantly associated with lipid metabolism and amino acid catabolism, whereas downregulated genes (629 genes; 92.4% of total DEGs) were primarily related to cholesterol metabolism, including cholesterol biosynthesis and sterol regulatory element-binding proteins (SREBP)-regulated genes (Fig. S3, Table S3 and S4).

Next, we analysed the transcriptional profile of chimeric murine hepatocytes (CMHs) in the same groups of chimeric mice. Upon WD feeding, mouse hepatocytes exhibited a total of 1,196 DEGs in comparison to NC-fed controls (Fig. 5C, Table S3 and S5). Notably, mouse DEGs showed little overlap with human DEGs. Among 87 upregulated mouse genes (7.3% of total DEGs), only 2 were also upregulated in human hepatocytes (*Arsb/ARSB* and *Fatp2/SLC27A2*) (Fig. 5D). Similarly, 1,109 mouse orthologues were shown to be downregulated (92.7% of total DEGs), from which only 92 genes were also identified among downregulated human orthologues (Fig. 5D). These findings indicate that, in the setting of hepatic steatosis, the mouse hepatocyte transcriptional signature markedly differs from the human counterparts.

### Alignment of cholesterol biosynthesis transcriptional networks between xenograft human hepatocytes and clinical NAFLD

**Fig 6:**
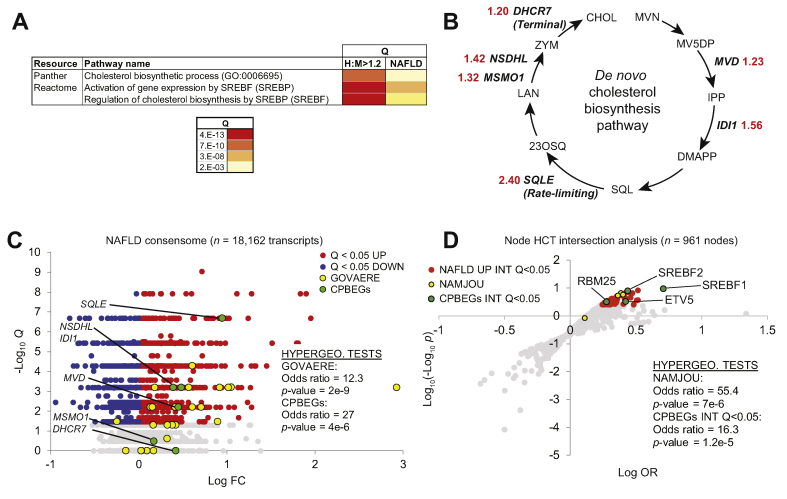
Distinct human and mouse cholesterol biosynthesis enzyme expression profiles in chimeric livers recapitulate clinical NAFLD. (A) Enrichment of SREBP1-cholesterol biosynthesis transcriptional pathways connects chimeric human hepatocytes (CHHs) and clinical NAFLD. (B) H:M >1.2 genes encode enzymes in the *de novo* cholesterol biosynthesis pathway. Enzyme names are shown in (D). Red numerals refer to CHH:CMH relative expression ratios. (C) The human NAFLD transcriptomic consensome ranks 18,162 genes based on their discovery rates across 20 independent, publicly archived clinical NAFLD case:control transcriptomic datasets. Hypergeometric test statistics for over-representation of the 25-gene NAFLD severity signature (GOVAERE) and CBPEGs among NAFLD CTs (mean case: control FC >1.25) are indicated. Q, FDR-corrected consensome *p*-value. (D) Node HCT intersection analysis of CPBEGs and FC >1.2 Q <0.05 clinical NAFLD consensome genes. NAFLD UP INT Q<0.05: nodes with significant (Q <0.05) intersections with NAFLD consensome CTs with mean case:control FC>1.2. CPBEGs INT Q <0.05: nodes with significant (Q <0.05) intersections with CPBEGs. Full data are in Table S6, section 4. CBPEG, cholesterol biosynthesis pathway enzyme genes; FC, fold change; FDR, false discovery rate; HCT, high confidence transcriptional target; NAFLD, non-alcoholic fatty liver disease; OR, odds ratio; SREBP1, sterol regulatory element-binding protein 1.

Given the abundant evidence connecting cholesterol metabolism to NAFLD,^28^ we next explored transcriptional mechanisms that might contribute to the marked rise in plasma and hepatic cholesterol levels observed in chimeric mice upon WD-feeding (Fig. 1E and Table S1). We generated a list of transcripts that were differentially expressed (*P* <0.05) in response to WD in both CHHs and CMHs, and had relative CHH:CMH expression levels that exceeded 20% (H:M>1.2; Table S6, section 1). Pathway analyses of this gene set reflected significant enrichment of genes mapped to cholesterol biosynthesis and SREBP-regulated gene expression (Fig. 6A). Closer inspection of the H:M>1.2 transcripts indicated that 6 represented *de novo* cholesterol biosynthesis pathway enzyme genes (CBPEGs; Fig. 6B and Table S6, sections 1 and 2). Notable among these were squalene epoxidase (*SQLE*), which catalyses the rate-limiting conversion of squalene to 2,3-oxidosqualene, and dehydrocholesterol reductase 7 (*DHCR7*), the terminal enzyme in the Kandutsch-Russell pathway for cholesterol synthesis. Although CBPEGs were repressed in both CMHs and CHHs in response to WD-feeding, this downregulation was much less efficient in CHHs, such that CBPEGs were expressed at levels up to 2.4-fold higher in CHHs relative to CMHs (Fig. 6B and Table S6, section 2).

We next evaluated the extent to which elevated expression levels of CBPEGs in the CHHs relative to the CMHs reflected clinical NAFLD. Using our previously-described consensome algorithm,^11^ we generated a clinical NAFLD transcriptomic consensome (Fig. 6C), which ranks ~18,100 human genes according to the frequency with which they are upregulated or downregulated across 22 independent published clinical NAFLD case-control transcriptomic datasets (Table S6, sections 2 and 3). Validating the NAFLD consensome, a 25-gene signature recently published by Govaere *et al.*^29^ that predicts clinical NAFLD severity was robustly enriched among mean FC>1.25 clinical NAFLD CTs (Q<0.05) in the consensome (GOVAERE: OR = 12.3, *p* = 2e-9; Fig. 6C and Table S6, section 2). Confirming the xenograft mouse as an accurate model of clinical NAFLD, we identified a very strong enrichment of the 6 CBPEGs (Fig. 6B) among the same set of clinical NAFLD CTs (CPBEGs: OR = 27, *p* = 4 e-6; Fig. 6C). Taken together, these data demonstrate a robust convergence between the elevated expression of CBPEGs in clinical NAFLD and in steatotic CHHs in the chimeric mice.

We next wished to gain insight into the extent to which transcriptional drivers of the xenograft model CPBEGs aligned with those implicated in human clinical NAFLD. To investigate this, we performed node HCT intersection analysis, which generates statistical measures of intersections between a given set of genes and HCTs for cellular signalling pathway nodes.^14^ For HCT intersection analysis, we computed intersections between the CPBEGs and NAFLD consensome case:control FC>1.2 CTs (NAFLD UP) and a library of signalling pathway node HCTs (Fig. 6D and Table S6, section 4). We benchmarked this analysis against a study by Namjou *et al.*^30^ that identified PPARG, FLI1, SPI1, and CEBPA as liver transcriptional drivers of a set of genes that had significant genome-wide association studies (GWAS) associations with NAFLD. Validating our HCT intersection analysis, 3 of these 4 nodes had NAFLD UP intersections of Q<1E-3 (NAMJOU: OR = 55.44, *p* = 7.6e-6; Fig. 6D and Table S6, section 4). We identified 4 nodes that had Q<0.05 intersections with the CPBEGs: SREBF1 (Q *=* 8e-10), SREBF2 (Q *=* 1e-4), ETV5 (Q *=* 8e-3) and RBM25 (Q *=* 5e-3). Again, reflecting close agreement between our model and clinical NAFLD, all 4 also had Q <0.05 intersections with NAFLD UP gene set (OR = 16.3, *p* = 1.2e-5, hypergeometric test; Fig. 6D and Table S6, section 4). SREBF1 and SREBF2 are both connected by abundant evidence to cholesterol biosynthesis^31^ and NAFLD pathogenesis.^32^ ETV5 is a transcriptional regulator of hepatic cholesterol transport and biosynthesis^33^ recently implicated in hepatic steatosis.^34^ Finally, a variant of RBM25 has been previously associated with familial hypercholesterolaemia.^35^ Collectively, our reduced-bias, consensus-driven approach reflects close convergence between cholesterol biosynthesis transcriptional regulatory networks in our xenograft model and those driving clinical NAFLD.

## Discussion

Our goal in this study was to establish and characterise a mouse model that faithfully replicated the pathophysiology of human NAFLD. Our humanised TIRF mice recapitulate histological, metabolic and transcriptional features of human NAFLD after 12 weeks on WD, with steatosis being confined in large to the human hepatocytes.

There were several similarities between lipid profiles in the NAFLD xenograft mice and patients with NAFLD. Free cholesterol and triacylglycerol lipid classes are increased both in livers of patients with NAFLD^21^^,^^28^ and NAFLD xenograft mice, as are hepatic concentrations of cholesteryl esters, which supports the role of these lipids in the pathogenesis of NAFLD. Multiple studies have suggested that PUFA depletion also contributes to the pathogenesis of human NAFLD.^27^ Obese NAFLD patients exhibit marked decreases in hepatic concentrations of n-3 PUFAs,^36^^,^^37^ and two mechanisms have been proposed to explain this observation: (i) an increased utilisation of n-3 PUFAs owing to the high oxidative status,^27^ and (ii) a reduced capacity for desaturation of α-linoleic acid (18:3n-3; a precursor of n-3 PUFAs) owing to decreases in hepatic activities of Δ-5 and Δ-6 desaturases.^38^ In line with this, livers of chimeric mice showed lower concentrations of PUFAs along with a trend towards downregulated expression of *FADS1* and *FADS2* genes, which encode human Δ-5 and Δ-6 desaturases, respectively. Alterations in hepatic PUFA content are thought to influence hepatic lipid homeostasis by regulating transcription factors such as PPARα and SREBP1c. Under normal circumstances, PUFAs act as activators of PPARα and inhibitors of SREBP1c, thereby promoting FA oxidation at the expense of lipogenesis, but in livers of obese patients with NAFLD, PUFA depletion correlates with reduced mRNA levels of *PPARA* and increased *SREBP1c*, potentially leading to a pro-lipogenic program that exacerbates the intrahepatic lipid accumulation in these patients.^39^ Although *PPARA* mRNA levels remained unchanged in the conditions tested in the present work, human liver chimeric mice recapitulated the increase in *SREBP1c* gene expression, further supporting the involvement of this transcription factor in early stages of human NAFLD.

A unique feature of NAFLD xenograft mice is the ability to discern transcriptional discrepancies between human and mouse hepatocytes exposed to identical nutritional challenges within chimeric livers. Illustrating this, we identified 6 CPBEGs that were highly repressed in response to WD in CMHs, but to a lesser extent in CHHs. Using a reduced-bias consensome meta-analysis of published NAFLD transcriptomic datasets, we demonstrated that the elevated expression of CBPEGs in CHHs relative to CMHs closely reflected the relative expression levels of their human orthologs between clinical NAFLD and normal livers. The gene with the highest CHH:CMH ratio (2.4), and the highest ranked CBPEG in the NAFLD consensome (23rd of 18,162 genes) was *SQLE*, whose expression has been connected to the pathogenesis of hepatic steatosis and its subsequent progression to hepatocellular carcinoma.^40^ Indeed, the identification of squalene expoxidase as a major mediator of increased hepatic biosynthesis of cholesteryl esters^40^ represents a plausible explanation for the elevated levels of these lipid species in livers of WD-fed chimeric mice. Finally, we identified robust evidence for convergence between transcriptional drivers of CPBEG expression and those regulating expression of genes consistently induced in clinical NAFLD.

In addition to its strengths, our NAFLD xenograft model has some limitations. Human albumin levels reveal a slight but significant decrease in humanisation after 12 weeks of WD feeding. Although this might constitute an experimental limitation particularly if animals do not have a high human liver chimerism, this observation is neither surprising nor diminishing the value of this NAFLD model. We did not apply selection for human hepatocytes in the murine liver during feeding experiments, and it is conceivable that steatotic human hepatocytes do not regenerate as efficiently as non-steatotic human hepatocytes in this chimeric mouse model. Although we do not provide direct evidence for this hypothesis, it is well established that liver regeneration is severely reduced in patients with NAFLD,^41^^,^^42^ which seems to translate to regeneration during homeostasis in this NAFLD xenograft model. Further, in the present study we used human hepatocytes from only three donors and human steaotosis is likely to depend on patient genotypes. For instance, it has been shown by GWAS that the single variant (rs738409, C>G, I148M) in the patatin-like-phospholipase domain-containing protein 3 (PNPLA3) gene is associated with increased liver fat content.^43^ This variant has a high prevalence in the Hispanic population and could also be found in 2 of our 3 donors (not shown). However, the third donor had two wild-type alleles in the PNPLA3 gene and developed the same degree of steatosis as the PNPLA3 I148M variants. To demonstrate a difference in steatosis between highly polymorphic human hepatocytes, many more donors are needed, which is outside of the scope of this study. However, as shown here this NAFLD xenograft model offers the possibility to use isogenic controls (same hepatocytes), which can compensate for the high degree of polymorphism in humans.

Another interesting observation was the lack of increase of body weight despite 12 weeks of WD feeding. Although we were unable to detect changes in hepatic markers of energy expenditure that could explain this phenotype, it is possible that the genetic background of mice impacts their susceptibility to diet-induced obesity.^44^^,^^45^ Nonetheless, NAFLD also occurs in lean individuals with a global prevalence ranging from 5 to 26%^46^ and non-obese patients with NAFLD share metabolic features with obese patients with NAFLD, including insulin resistance, dyslipidaemia, and metabolic syndrome, and there is little appreciable difference in NAFLD progression between those groups.^46^^,^^47^ Our NAFLD mice are also immunodeficient and are unlikely to develop the inflammatory changes or fibrosis that lead to NASH. In addition, current technology does not allow efficient repopulation of human non-parenchymal cells, which would be necessary for modelling fibrosis. Notwithstanding, our humanised NAFLD mice unite the experimental tractabilty of the mouse with the translational value of human hepatocytes. Our NAFLD model will be useful for mechanistic studies on the molecular basis of the disease, as well as preclinical testing of experimental therapies, thereby enabling the discernment of cellular processes that are truly relevant to human NAFLD.

## Financial support

Supported by the 10.13039/100000050National Heart Lung and Blood Institute (R01 HL134510 to K.D.B. and R01 HL132840 to W.R.L.), 10.13039/100000062National Institute of Diabetes and Digestive and Kidney Disease (R01 DK115461 to K.D.B.; R01 DK056626, R01 DK103046, and R37 DK048872 to D.E.C.; DK097748 to N.J.M.) and the 10.13039/100000968American Heart Association (18POST33990445 and 20CDA35340013 to M.A.-B.). The TEAM programme of the Foundation for Polish Science co-financed by the European Union under the 10.13039/501100008530European Regional Development Fund to M.B., and POLONEZ grant from National Science Center (2015/19/P/NZ3/03452) and UE Horizon 2020 (MSCA 665778) to M.B

## Authors’ contributions

Designed the experiments: K.D.B., B.B.C. Primary data analysis: B.B.C., M.A.-B. Performed *in vivo* experiments: B.B.C., Me. B.Tissue isolation and preparations: X.L. Prepared libraries and run sequencing: D.Y., Ma. B. Assisted with hepatocyte handling and procurement: A.M.D., N.T.N.G., R.B.Y., J.G. Bioinformatic analysis: M.A.-B., B.Z., S.A.O., N.J.M., P.S. Assisted with data interpretation and presentation: W.R.L., D.D.M., D.E.C. Read and approved the final manuscript: all authors

## Data availability statement

RNA-Seq data are available from the European Nucleotide Archive, ENA accession code (PRJEB35014).

## Conflicts of interest

The authors declare no personal or financial conflicts of interest.

Please refer to the accompanying ICMJE disclosure forms for further details.
