# Supplementary material for: A human liver chimeric mouse model for non-alcoholic fatty liver disease
Source: JHEP Rep. 2021 Mar 21;3(3):100281. doi: 10.1016/j.jhepr.2021.100281 (PMC8138774; doi:10.1016/j.jhepr.2021.100281)
Supplement: Multimedia component 1 [file mmc1.pdf]

A human liver chimeric mouse model for non-alcoholic fatty liver disease

Beatrice Bissig-Choisat, Michele Alves-Bezerra, Barry Zorman, Scott A. Ochsner, Mercedes Barzi, Xavier Legras, Diane Yang, Malgorzata Borowiak, Adam M. Dean, Robert B York, N. Thao N. Galvan, John Goss, William R. Lagor, David D. Moore, David E. Cohen, Neil J. McKenna, Pavel Sumazin, Karl-Dimiter Bissig

Table of contents

Supplementary methods....................2

Fig. S1....................10

Fig. S2....................11

Fig. S3....................12

Table S3....................13

Supplementary references....................14

Supplementary methods

Blood chemistry

Mouse blood was collected by retro-orbital puncture. Serum levels of ALT, AST, ALP, GGT, TAG and total cholesterol were measured using an AU480 Chemistry Analyzer (Beckman Coulter).

Liver histology, immunohistochemistry and quantification of steatotic liver

Liver samples were fixed with 4% paraformaldehyde overnight for paraffin blocks or embedded in blocks using optimal cutting temperature compound (Fisher). Paraffin sections were used either for hematoxylin and eosin (H&E) or Mason Trichrome staining using standard techniques. For immunostaining, paraffin sections were incubated with primary antibodies - F4/80 (MCA497, Bio-Rad) diluted 1:250 or FAH (sc-66223, Santa Cruz Biotechnology) diluted 1:500. Then, biotinylated secondary antibody was incubated for 30 min. Staining was performed using the M.O.M Vectastain kit and DAB detection kit (Vector Laboratories).

Images of FAH immunostaining were taken, and composite images of each lobe (Adobe Photoshop) were used to determine total area of FAH positive cells (ImageJ software by W. Rasband, National Institutes of Health, Bethesda, MD; http://rsb.info.nih.gov/ij). Percentage of areas with micro- respectively macrovesicular steatosis as well as non-steatotic areas were calculated (ImageJ software) for each lobe. The same calculations were done for FAH negative areas (murine cells). Total liver lobe area was used to validate proper calculations: FAH^{+}normal + FAH^{+}microvesicular +

FAH^+macrovesicular + FAH^-normal + FAH^-microvesicular + FAH^-macrovesicular = total liver lobe area.

Global metabolomic profiling

Sample preparation was carried out at Metabolon Inc. (Durham, NC). The global biochemical profiling analysis comprised four arms: (i) reverse phase chromatography positive ionization methods optimized for hydrophilic compounds (LC/MS Pos Polar); (ii) hydrophobic compounds (LC/MS Pos Lipid); (iii) reverse phase chromatography with negative ionization conditions (LC/MS Neg); and (iv) HILIC chromatography method coupled to negative (LC/MS Polar). All of the methods alternated between full-scan MS and data-dependent MSn scans (70–1000 m/z). Metabolites were identified by automated comparison to a reference library of chemical standard entries that included retention time, molecular weight (m/z), preferred adducts, and in-source fragments as well as associated Identification of known chemical entities was based on comparison to metabolomic library entries of purified standards.

Untargeted lipidomics

Lipids were extracted from snap-frozen liver samples at Metabolon Inc. using dichloromethane and methanol in the presence of deuterated internal standards. The organic phase was dried under a N_2 stream and reconstituted in dichloromethane:methanol (1:1) containing 10 mM ammonium acetate. Flow injection and mass spectrometry (FIA-MS) was performed on a Sciex 5500 QTRAP equipped with a SelexION Differential Mobility Separation (DMS) cell and operated in Multiple Reaction

Monitoring (MRM) mode using both positive and negative mode electrospray in a Turbo V ion source.

Each sample was subjected to two analyses. In the first analysis, we monitored 472 MRM pairs, corresponding to 448 endogenous lipids and 24 internal standards. The SelexION was used to apply Compensation Voltages (CoV) optimized for each lipid class, using n-propanol as the DMS modifier and the Separation Voltage set to 3500 V. In the second analysis, we monitored 706 MRM pairs corresponding to 676 endogenous lipids and 30 internal standards, and the SelexION was not used. Both analyses included 20 MRM cycles with 20 msec per MRM pair, a settling time of 50 msec, and a pause between mass ranges of 5 msec. Individual lipid species were quantified based on the ratio of signal intensity for target compounds to the signal intensity for an assigned deuterated internal standard of known concentration. Lipid class concentrations were calculated from the sum of all molecular species within a class, and fatty acid compositions were determined by calculating the proportion of individual fatty acids within each class.

RNA-Sequencing

Whole-transcriptome RNA sequencing (RNA-Seq) was performed using total RNA isolated using Purelink RNA mini kit (Invitrogen). Libraries were generated using TrueSeq Stranded mRNA LT kit (Illumina, Hayward, CA) and sequenced on a NextSeq 500 sequencer (Illumina). RNAseq read pairs were initially aligned to a combined human (hg38) and murine (mm10) genome using STAR version 2.7.1a. Secondary alignments were removed, and reads were filtered by alignment to human and mouse components using SAMtools version 1.9. BEDtools version 2.20.1bamtofastq was used to extract

reads from the assigned species bam files. Extracted read pairs were input for transcript and gene quantification with RSEM version 1.3.0 using the read aligner Bowtie2 to the corresponding NCBI Refseq transcriptome (3/21/2016) of each species. Gene expression was normalized to four human housekeeping genes and their murine counterparts (*PSMB2*, *PSMB4*, *RAB7A*, and *VPS29*; *Psmb2*, *Psmb4*, *Rab7*, and *Vps29*) [1]. Low-abundance transcripts [< 5 (housekeeping-normalized) TPM for the averages of both dietary phenotypes] were excluded from the analysis. RNA-Seq data is available from European Nucleotide Archive, ENA accession code (PRJEB35014). Pathway-enrichment analysis was performed using Enrichr for the Reactome Pathways database (version 2016), TRRUST transcriptional regulatory interactions database (version 2019), and miRTarBase microRNA-target interactions database (version 2017).

NAFLD consensome analysis

To generate the human NAFLD consensome, we first retrieved a total of nine Gene Expression Omnibus (GEO) series in which clinical liver biopsies from NAFL or NASH patients and healthy individuals were committed to expression array or RNA-Seq analysis (Suppl. Table 6, section 3). Sample files were organized into case versus control contrasts.

To process microarray expression data, we utilized the log2 summarized and normalized array feature expression intensities provided by the investigator and housed in GEO. These data are available in the corresponding “Series Matrix Files(s)”. The full set of summarized and normalized sample expression values were extracted and processed using R software (http://cran.r-project.org). To calculate differential gene

expression for investigator-defined experimental contrasts, we used the linear modeling functions from the Bioconductor limma analysis package [2]. Initially, a linear model was fitted to a group-means parameterization design matrix defining each experimental variable. Subsequently, we fitted a contrast matrix that recapitulated the sample contrasts of interest, in this case viral infection versus mock infection, producing fold-change and significance values for each array feature present on the array. The current BioConductor array annotation library was used for annotation of array identifiers. P values obtained from limma analysis were not corrected for multiple comparisons.

To process RNA-Seq expression data, we utilized the aligned, un-normalized, gene summarized read count data provided by the investigator and housed in GEO. These data are available in the corresponding “Supplementary file” section of the GEO record. The full set of raw aligned gene read count values were extracted and processed in the statistical program R using the limma and edgeR analysis packages [3]. Read count values were initially filtered to remove genes with low read counts. Gene read count values were passed to downstream analysis if all replicate samples from at least one experimental condition had cpm > 1. Sequence library normalization factors were calculated to apply scale normalization to the raw aligned read counts using the TMM normalization method implemented in the edgeR package followed by the voom function126 to convert the gene read count values to log2-cpm. The log2-cpm values were initially fit to a group-means parameterization design matrix defining each experimental variable. This was subsequently fit to a contrast design matrix that recapitulates the sample contrasts of interest (case versus control contrasts) producing fold-change and significance values for each aligned sequenced gene. If necessary, the

current BioConductor human organism annotation library was used for annotation of investigator-provided gene identifiers. P values obtained from limma analysis were not corrected for multiple comparisons.

Differential expression values were committed to the consensome analysis pipeline as previously described [4]. Briefly, the consensome algorithm surveys each experiment across all datasets and ranks genes according to the frequency with which they are significantly differentially expressed. For each transcript, we counted the number of experiments where the significance for differential expression was ≤0.05, and then generated the binomial probability, referred to as the consensome p-value (CPV), of observing that many or more nominally significant experiments out of the number of experiments in which the transcript was assayed, given a true probability of 0.05. P values were adjusted using the p.adj function in R to generate q values (CQV). Genes were ranked firstly by CQV, then by mean fold change (MFC). The consensomes and underlying datasets were loaded into an Oracle 15c database and made available on the SPP user interface as previously described [4].

High confidence transcript intersection analysis

High confidence transcript intersection analysis [5]was used to compute overlap between a given gene list and SPP ChIP-Seq consensome sets of 960 human or 696 mouse signaling pathway nodes. Genes in the top 5% of these consensomes were designated as high confidence transcriptional targets (HCTs) for a given node. Briefly, we used the Bioconductor GeneOverlap analysis package implemented in R. Given a whole set I of IDs and two sets $A \in I$ and $B \in I$, and $S = A \cap B$, GeneOverlap calculates the

significance of obtaining S. The problem is formulated as a hypergeometric distribution or contingency table, which is solved by Fisher's exact test. P values were adjusted for multiple testing by using the method of Benjamini & Hochberg to control the false discovery rate as implemented with the p.adjust function in R, to generate q values. The universe for the intersection was set at a conservative estimate of the total number of transcribed (protein and non-protein-coding) genes in the human (25,000) or mouse (30,000) genomes.

Literature-based pathway analysis

Panther [6] or Reactome pathway [7] analysis was used to evaluate enrichment of biological processes in NAFLD consensome confidence DEGs (FC > 1.25, $q < 0.05$) or genes whose expression levels in chimeric human (CHHs) in response to WD exceeded those in chimeric mouse hepatocytes (CMHs) in response to WD by more than 20% (H:M>1.2).

Quantification and Statistical Analyses

Statistical significance was determined using the two-tailed unpaired Student *t* test for comparisons between two groups, or one-way ANOVA followed by Tukey's post-test for comparisons among three groups. Statistical analyses, including NAFLD consensome hypergeometric tests, were performed using GraphPad Prism 7 (GraphPad Software, San Diego, CA). For metabolome and lipidome analysis, standard statistical analyses were performed in Array Studio (Omicsoft) on log-transformed data. For non-standard analyses, we used R software (http://cran.r-project.org). Following log transformation and

imputation of missing values, if any, with the minimum observed value for each compound, we used Welch's two sample *t*-Test for comparisons between groups. Differences were considered significant at P less than 0.05 and false discovery rate (q-value) higher than 0.25. Metabolite and lipid set enrichment was implemented using the GSEA Program 3.0 [8] with the Metabolon metabolite/lipid sub-pathways used as the set reference. Runs were performed with 10,000 randomized metabolite/lipid sets for statistical significance estimation and the signal-to-noise metric (Z-score) between the two phenotypes was used to rank all metabolites/lipids. Sub-pathway sets with fewer than 3 or more than 500 molecules were excluded from the enrichment analysis. Statistical significance for transcriptomic and consensomes was determined as described [4]. Linear regression analysis was carried out using the Data Analysis Correlation function in Microsoft Excel 2016.

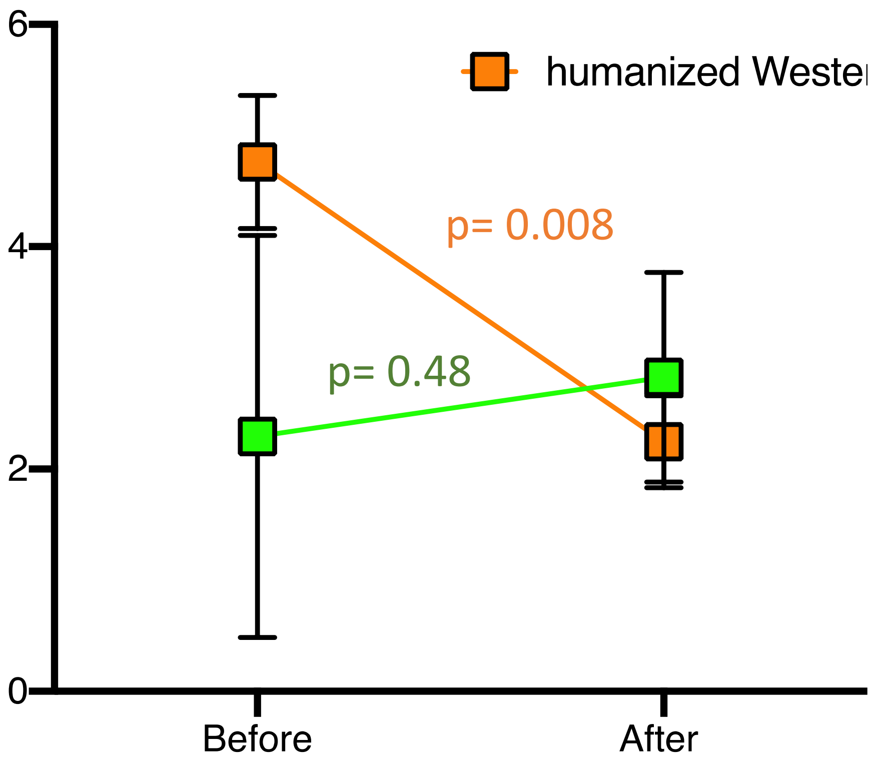

Fig. S1. Repopulation rates of human liver chimeric mouse as measured by human albumin in the murine blood. Normal chow (NC) or Western-type diet (WD) (n = 4/group). P-values of Mann-Whitney test comparing before and after diet are given for both groups.

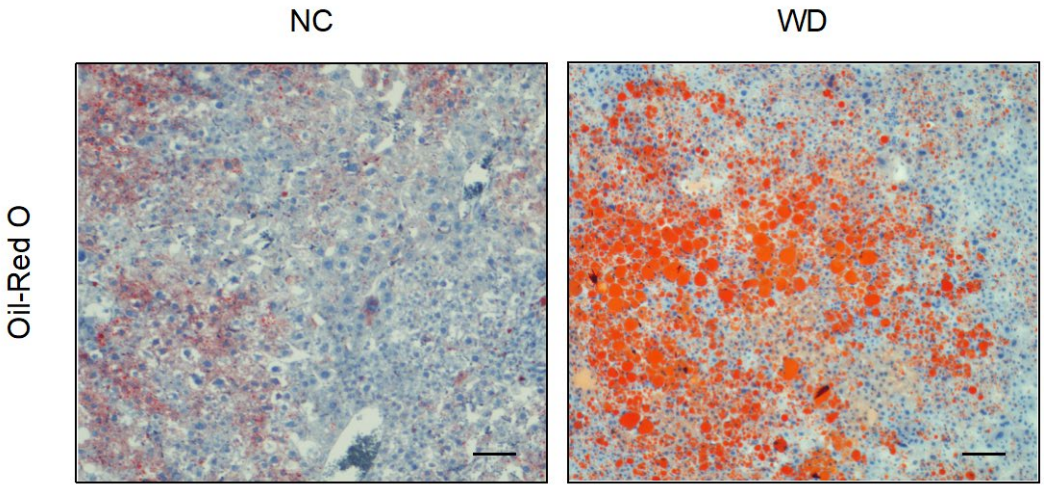

Fig. S2. Diet-induced steatosis in NAFLD xenograft mice. Representative images of Oil-Red O-stained liver sections obtained from the humanized mice after 12 w of normal chow (NC) or Western-type diet (WD) (n = 6-8/group). Scale bar 50 μm.

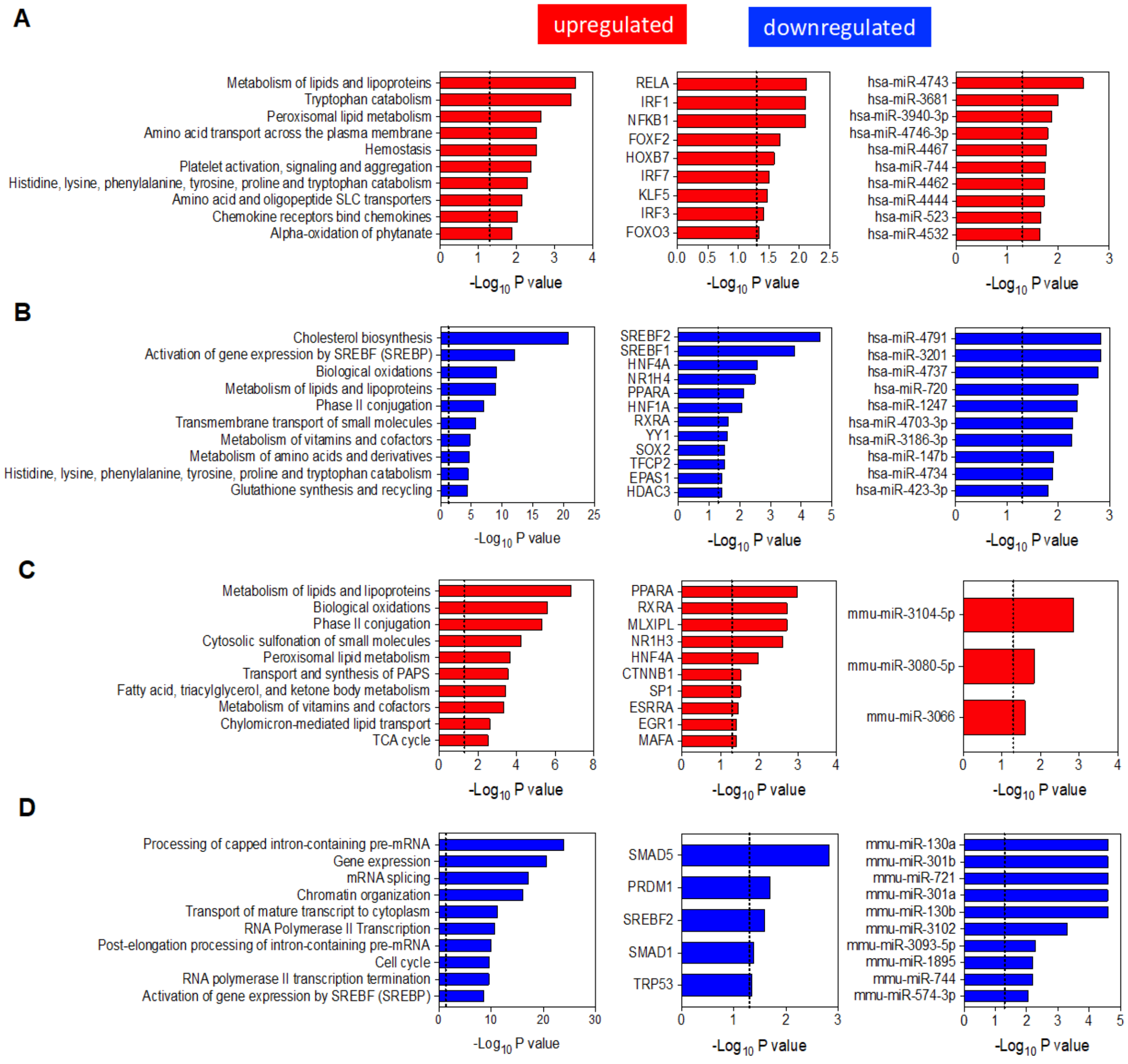

Fig. S3. NAFLD xenograft mice undergo changes in hepatic transcriptional program. Transcriptome analysis of human (A and B) and mouse (C and D) orthologues was performed in livers of humanized mice after 12 w of either normal chow (NC) or Western-type diet (WD) (n = 8/group). Gene set enrichment analysis was performed for upregulated (A and C) and downregulated (B and D) genes: left panels, Reactome pathways; Middle panels, TRRUST Transcription Factors; Right panels, TargetScan microRNA.

Table S3. Top differentially regulated genes in NAFLD xenograft mice

**Gene Name**	**Pathways[a,b] (SuperPathway[c])**	***P* value**	**Fold change (NAFLD vs control[d])**
Upregulated			
ARSB (Arylsulfatase B)	Immune system[a]<br>Chondroitin sulfate/dermatan sulfate metabolism[a]<br>Glycosaminoglycan metabolism[a]<br>Metabolic pathways[b]	2.13E-02	2.01
BCL2L11 (BCL2-Like Protein 11)	Intrinsic Pathway for Apoptosis[a]<br>p75 NTR receptor-mediated signaling[a]<br>Apoptosis<br>(Activation of BH3-only proteins, Apoptosis Modulation and Signaling)[c]	6.02E-03	1.69
CXCL10 (Small Inducible Cytokine Subfamily B, Member 10)	GPCR ligand binding<br>IL-17 signaling pathway<br>TNF signaling pathway	2.16E-02	2.51
DESI2 (Desumoylating Isopeptidase 2)	NA	7.61E-03	2.08
FAM156A (Transmembrane Protein 29)	NA	1.33E-02	1.83
FOXO1 (Forkhead Box Protein O1)	Signaling by PDGF [a]<br>MAPK family signaling cascades<br>PIP3 activates AKT signaling<br>(RET signaling, PI3K/AKT activation)[c]	9.45E-03	1.67
LMF1 (Lipase Maturation Factor 1)	Lipid digestion, mobilization, and transport<br>metabolism of lipids and lipoproteins[a]	1.28E-02	2.39

PDGFA<br>(Platelet Derived Growth Factor Alpha Polypeptide)	RAF/MAP kinase cascade<br>Negative regulation of the PI3K/AKT network<br>Choline metabolism in cancer<br>(RET signaling, PI3K/AKT activation)[c]	4.31E-02	1.67
PWWP2B<br>(PWWP Domain Containing 2B)	NA	1.66E-02	1.79
RAB11FIP5<br>(RAB11 Family Interacting Protein 5)	Endocytosis[b]	1.57E-02	2.07
RAB28<br>(RAB28, Member RAS Oncogene Family)	NA	2.93E-02	1.70
SECTM1<br>(Secreted and Transmembrane 1)	NA	6.77E-03	2.81
SLC7A10<br>(Solute Carrier Family 7 Member 10)	Transmembrane transport of small molecules[a] (transport of glucose and other sugars, bile salts and organic acids, metal ions and amine compounds)[c]	2.07E-02	8.23
SREBF1<br>(Sterol Regulatory Element Binding Transcription Factor 1)	Activation of gene expression by SREBP<br>AMPK signaling pathway<br>(Regulation of lipid metabolism by PPAR-alpha, Regulation of cholesterol biosynthesis by SREBP)[c]	5.61E-03	1.79
TDO2<br>(Tryptophan 2,3-Dioxygenase)	Tryptophan catabolism<br>Metabolic pathways[b]	3.08E-02	1.85
Downregulated			
CDKN2C<br>(Cyclin Dependent Kinase Inhibitor 2C)	Cell Cycle<br>Mitotic[a]<br>Oxidative Stress Induced Senescence	1.88E-02	0.40

	Oncogene Induced Senescence[a]		
CTGF (CCN2)<br>(Cellular Communication<br>Network Factor 2)	Fatty acid, triacylglycerol and ketone body metabolism<br>metabolism of lipids and lipoproteins<br>Hippo signaling pathway[b]	1.12E-02	0.29
CYP1A1<br>(Cytochrome P450 Family 1, Subfamily A Member 1 (Cholesterol 25-Hydroxylase)	Oxidation by Cytochrome P450<br>Arachidonic acid metabolism<br>Metabolism of lipids and lipoproteins<br>Chemical carcinogenesis[b]<br>Retinol metabolism[b]	5.86E-03	0.09
CYP1A2<br>(Cytochrome P450 Family 1 Subfamily A Member 2) (Cholesterol 25-Hydroxylase)	Drug metabolism-cytochrome P450<br>Metabolism of lipids and lipoproteins<br>Chemical carcinogenesis[b]	3.55E-04	0.09
FAM156B<br>(Transmembrane Protein 29B)	NA	2.11E-02	0.33
HAMP<br>(Hepcidin Antimicrobial Peptide, Liver-Expressed Antimicrobial Peptide)	TGF-Beta signaling pathway[b]	7.87E-03	0.13
HAUS5<br>(HAUS Augmin Like Complex Subunit 5)	Cell Cycle<br>Mitotic<br>Centrosome maturation<br>(Regulation of PLK1 Activity at G2/M Transition)[c]	4.29E-02	0.38
HMGCR<br>(3-Hydroxy-3-Methylglutaryl-CoA Reductase; NADPH)	Cholesterol biosynthesis[a]<br>Fatty acid, triacylglycerol, and ketone body metabolism<br>AMPK signaling pathway<br>Bile secretion[b]	4.89E-04	0.40
HMGCS1<br>(3-Hydroxy-3-Methylglutaryl-CoA	Cholesterol biosynthesis<br>Fatty acid, triacylglycerol, and	4.37E-03	0.41

Synthase 1)	ketone body metabolism[a]<br>(Regulation of lipid metabolism by PPAR-alpha, Regulation of cholesterol biosynthesis by SREBP)[c]		
LOC101929950<br>(Puromycin-Sensitive Aminopeptidase-Like Protein)	NA	2.32E-02	0.39
RAB26<br>(Ras-Related Oncogene Protein)	Metabolism of proteins	8.14E-03	0.36
SFT2D2<br>(Vesicle Transport Protein SFT2B)	NA	4.86E-02	0.35
SUGP2<br>(Arginine/Serine-Rich-Splicing Factor 14)	mRNA Processing	3.59E-03	0.41
TKFC<br>(Triokinase and FMN Cyclase)	Fructose catabolism and metabolism<br>Immune System<br>Metabolism of carbohydrates[b]	2.31E-02	0.37
TM7SF2<br>(Transmembrane 7 Superfamily Member 2)	Cholesterol biosynthesis<br>Activation of gene expression by SREBP<br>Metabolic pathways<br>(Regulation of cholesterol biosynthesis by SREBP)[c]	1.51E-03	0.42

[a]Reactome pathway
[b]KEGG (Kyoto Encyclopedia of Genes and Genomes) pathway
[c]SuperPathways
[d]Humanized mice on WD (NAFLD) compared to humanized mice on normal chow (control)
NA, not assigned

Supplementary references

[1] Eisenberg E, Levanon EY. Human housekeeping genes, revisited. Trends in genetics : TIG 2013;29:569-574.

[2] Ritchie ME, Phipson B, Wu D, Hu Y, Law CW, Shi W, et al. limma powers differential expression analyses for RNA-sequencing and microarray studies. Nucleic Acids Res 2015;43:e47.

[3] Robinson JT, Thorvaldsdottir H, Winckler W, Guttman M, Lander ES, Getz G, et al. Integrative genomics viewer. Nat Biotechnol 2011;29:24-26.

[4] Ochsner SA, Abraham D, Martin K, Ding W, McOwiti A, Kankanamge W, et al. The Signaling Pathways Project, an integrated 'omics knowledgebase for mammalian cellular signaling pathways. Sci Data 2019;6:252.

[5] Ochsner SA, Pillich RT, McKenna NJ. Consensus transcriptional regulatory networks of coronavirus-infected human cells. Sci Data 2020;7:314.

[6] Mi H, Huang X, Muruganujan A, Tang H, Mills C, Kang D, et al. PANTHER version 11: expanded annotation data from Gene Ontology and Reactome pathways, and data analysis tool enhancements. Nucleic Acids Res 2017;45:D183-D189.

[7] Fabregat A, Sidiropoulos K, Garapati P, Gillespie M, Hausmann K, Haw R, et al. The Reactome pathway Knowledgebase. Nucleic Acids Res 2016;44:D481-487.

[8] Subramanian A, Tamayo P, Mootha VK, Mukherjee S, Ebert BL, Gillette MA, et al. Gene set enrichment analysis: a knowledge-based approach for interpreting genome-wide expression profiles. Proc Natl Acad Sci U S A 2005;102:15545-15550.
