# Supplementary material for: A human liver chimeric mouse model for non-alcoholic fatty liver disease
Source: JHEP Rep. 2021 Mar 21;3(3):100281. doi: 10.1016/j.jhepr.2021.100281 (PMC8138774; doi:10.1016/j.jhepr.2021.100281)
Supplement: Multimedia component 8 [file mmc8.pdf]

JHEP Reports

CTAT methods

Tables for a “Complete, Transparent, Accurate and Timely account” (CTAT) are now mandatory for all revised submissions. The aim is to enhance the reproducibility of methods.

- Only include the parts relevant to your study
- Refer to the CTAT in the main text as ‘Supplementary CTAT Table’
- Do not add subheadings
- Add as many rows as needed to include all information
- Only include one item per row

If the CTAT form is not relevant to your study, please outline the reasons why:

1.1 Antibodies

Name	Citation	Supplier	Cat no.	Clone no.
F4/80	Tarallo V. et al. 2011 J Biol Chem. 286: 19641-51.10. Rivollier A. et al. 2012 J Exp Med. 209:139-55.	Bio-Rad	MCA49	clone CI:A3-1
FAH	Bergeron, A., et al. 2006. J. Biol. Chem. 281: 5329-5334. 5. Jacobs, S.M., et al. 2006. Pediatr. Res. 59: 365-370.	Santa Cruz Biotechnology	sc-66223	C-20

1.2 Cell lines

Name	Citation	Supplier	Cat no.	Passage no.	Authentication test method
none					

1.3 Organisms

Name	Citation	Supplier	Strain	Sex	Age	Overall n number
TIRF mouse	Barzi M. et al. 2017 Nat Commun. 2017 Oct 17;8(1):984.	Bissig lab	TIRF	M&F	any	Breeding and repopulation: n=50

1.4 Sequence based reagents

Name	Sequence	Supplier

1.5 Biological samples

Description	Source	Identifier
Human hepatocytes	**TRL**	**HUM4043**

1.6 Deposited data

Name of repository	Identifier	Link
European Nucleotide Archive	PRJEB35014	https://www.ebi.ac.uk/ena/browser/home

1.7 Software

Software name	Manufacturer	Version
GraphPad Prism	GraphPad Software	7
Array Studio	Omicsoft	1
R software	R Foundation	3
Samtools	Genome Research Limited	1
GSEA software	UC San Diego; Broad Institute	3
Excel	Microsoft	2016

1.8 Other (e.g. drugs, proteins, vectors etc.)

1.9 Please provide the details of the corresponding methods author for the manuscript:

Karl-Dimiter Bissig, Duke University, 905 South LaSalle St., Durham NC-27708

2.0 Please confirm for randomised controlled trials all versions of the clinical protocol are included in the submission. These will be published online as supplementary information.

N/A
